# Comparison of two instruments for hypertension self‐care assessments among older adults from China

**DOI:** 10.1002/nop2.1422

**Published:** 2022-12-22

**Authors:** Qiao Zhao, Yujie Guo, Lipei Gu, Lei Yang

**Affiliations:** ^1^ Department of Nursing Wuxi Higher Health Vocational Technology School Wuxi China; ^2^ School of Medicine (School of Nursing) Nantong University Nantong China; ^3^ Department of Burn First People's Hospital of Nantong Nantong China; ^4^ Nursing Department Jiangsu Province Hospital Jiangsu Nanjing China

**Keywords:** hypertension self‐care, Hypertension Self‐Care Profile (HBP SCP), older adults, reliability, Self‐Care of Hypertension Inventory (SC‐HI), sensitivity, specificity, validity

## Abstract

**Aim:**

To test and compare the Self‐Care of Hypertension Inventory (SC‐HI) and Hypertension Self‐Care Profile (HBP SCP) among older patients with hypertension in China.

**Design:**

A cross‐sectional observational study.

**Methods:**

A convenience sampling of 220 older adults (120 male patients and 100 female patients) with hypertension and a mean age of 73.74 years was surveyed using the Chinese version of SC‐HI, the Chinese version of HBP SCP and the Chinese version of Exercise of Self‐Care Agency Scale (ESCA) during July‐September 2019. Psychometric analyses and Receiver Operating Characteristic curve analyses were performed on the collected data.

**Results:**

The Cronbach's *α* of SC‐HI and HBP SCP was 0.858 and 0.953, respectively. The Pearson's coefficients between the SC‐HI total score and the ESCA total score, the HBP SCP total score and the ESCA total score were 0.494 and 0.700, respectively. The satisfactory sensitivity, specificity, cut‐off point and Area under the curve of SC‐HI were 0.8292, 0.5495, 120.5 and 0.754, respectively. As for HBP SCP, the values were 0.7907, 0.7582, 169.5 and 0.838, respectively. There was no significant difference between these two scales. Each has its own characteristics. However, the HBP SCP is more precise and effective for measuring self‐care ability in older patients.

## 1 INTRODUCTION

Hypertension (defined as a health condition with either blood pressure >140/90 mmHg or individuals' self‐reported use of antihypertensive medication in the previous 2 weeks) (Hesketh & Zhou, 2017) is a common morbidity and a major risk factor for cardiovascular disease and stroke, accounting for 2.5 million deaths (27.5% of total deaths) and 14.28% of total disability‐adjusted life‐years in 2013 in China (Banegas, 2017; Yang et al., 2021). Various genetic and environmental factors lead to the incidence and persistence of hypertension (Banegas, 2017). In China, hypertension prevalence has risen in recent decades and increases with age (Hansell et al., 2017; Yang et al., 2021). Only 5.1% of the older population, who are more prone to target‐organ damage, report that their blood pressure is under control (Liew et al., 2019). The evidence suggests that public awareness and control of blood pressure remain a challenge (Lu et al., 2017). Poor control and lack of hypertension awareness will often seriously affect a patient's quality of life, especially among older adults.

Self‐care, a positive organizational behaviour characteristic, plays an indispensable role in disease prevention and management (Yang et al., 2022). As an important intervention to maintain health and manage chronic diseases, self‐care has been confirmed to be an effective method for the secondary prevention of hypertension and has been documented as one of the main determinants of hypertension control (Chobanian et al., 2003; Putri et al., 2021; Riegel et al., 2012; Tadesse & Gerensea, 2021). Cumulative evidence shows that hypertension self‐care is effective in controlling blood pressure and preventing complications related to hypertension (Ma, 2018).

Therefore, it is important to focus on the self‐care ability of older adults with hypertension. At present, self‐care assessment tools aimed at patients with hypertension are mainly used in clinical settings to measure hypertension self‐care ability, and targeted interventions are implemented to improve patients' self‐care ability accordingly (Chen et al., 2014). In view of this, it is necessary to develop reliable and valid instruments for measuring hypertension self‐care ability with regard to both clinical nursing practice and personal health. This could contribute to advances in hypertension self‐care research (Tomstad et al., 2013).

A variety of instruments have been used to assess the ability to self‐care, which allowed us to evaluate the adherence to antihypertensive medications, lifestyle modifications and self‐monitoring of blood pressure among older adults with hypertension and offer targeted interventions based on the individual's own perception of their general health and self‐care condition accurately in time. Further details are presented in Table 1 (Dong et al., 2017; Olle, 2009; Qiuli, 2012; Sousa et al., 2010; Warren‐Findlow et al., 2013). Although several self‐care assessment tools are used in older adults with hypertension, they have some limitations. For example, the Hypertension Patients Self‐Management Behaviour Rating Scale (HPSMBRS) only focuses on the self‐management behaviour of patients with hypertension. The Self‐Management Scale for Patients with Hypertension includes items on treatment management, diet and exercise, lifestyle and risk factor management, but it does not consider items on self‐efficacy or confidence in the management of hypertension. The Hypertension Self‐Care Activity Level Effects (H‐SCALE) are suitable for large‐scale epidemiological investigations. The Exercise of Self‐Care Agency Scale (ESCA), the Self‐Care Ability Scale for the Elderly (SASE) and the Appraisal of Self‐Care Agency Scale‐Revised (ASAS‐R), as generic scales, lack disease specificity. SASE and ASAS‐R are not as widely used in Chinese hypertensive patients as ESCA (Chen et al., 2014; Guo et al., 2017; Sousa et al., 2010; Zhao et al., 2019). Among these self‐care ability assessment scales, two specific valid scales are available to measure self‐care ability in patients with hypertension: the Self‐Care of Hypertension Inventory (SC‐HI) and the Hypertension Self‐Care Profile (HBP SCP), which have both been translated into Chinese and tested among patients with hypertension (Chen et al., 2014; Dickson et al., 2017; Han, Lee, et al., 2014; Koh et al., 2016; Ma et al., 2021; Seow et al., 2017; Sha et al., 2017; Silveira et al., 2018; Zhao et al., 2019). The items in these two scales cover behaviour, self‐management, self‐efficacy, confidence and so on. Moreover, both these scales have been developed based on the practice of self‐care health promotion and thus can effectively measure the self‐care ability of hypertensive patients in all aspects (Orem, 1985; Riegel et al., 2012). Additionally, during the process of adapting these two scales to the Chinese culture, the designation of each dimension was changed owing to the Chinese translation. Nonetheless, there are similar items and survey purposes in three dimensions of these two scales, making the results highly comparable to a certain extent. However, a comparative analysis of the effects of these two instruments has not yet been conducted among older adults with hypertension in China.

**TABLE 1:** Self‐report instruments used to measure self‐care ability of patients with hypertension

Instrument name	First author, nationality	No. dimensions, No. items	Content areas	Validity	Reliability	Target population
HPSMBRS	Zhao (Qiuli, 2012), China	6, 33	Diet, exercise, medication taking, mood management, lifestyle and disease management	Content validity index, 0.91; Construct: six factors (58.543% explained variance) CFA with a good model fit (NFI, 0.902; CFI, 0.956; RMSEA, 0.042)	relation:0.180–0.525; Item‐total correlation:0.579–0.830; Internal consistency: Cronbach's *α* = 0.914	Patients with hypertension
Self management scale for patients with hypertension	Liu (Dong et al., 2017), China	4, 21	Treatment management, diet and exercise, lifestyle, risk factor management	Content validity index, 0.976; Construct: 5‐factor model explaining 61.889% of variance	Internal consistency: Cronbach's *α* = 0.854; Guttman Split‐Half coefficient = 0.856	Patients with hypertension
H‐SCALE	Findlow (Warren‐Findlow et al., 2013), America	6, 32	Medication, weight management, physical activity, tobacco exposure, alcohol intake, DASH diet	Correlation: medication adherence was significantly correlated with systolic BP (*r* = −.19); DASH diet scores were associated with higher systolic BP (*r* = .19); weight managemen*t* scores were significantly correlated with lower diastolic BP (*r* = −.22)	Internal consistency: Cronbach's *α* = 0.67–0.88	Adults with hypertension
SASE	Olle Söderhamn (Olle, 2009), Sweden	3, 17	Repertoire, environment and goal	Construct: the final three‐factor model had an acceptable goodness‐of‐fit (2 = 198.60, df = 119) with RMSEA = 0.070	Item‐total correlation:0.28–0.39 Internal consistency: Cronbach's alpha = 0.68	Older adults
ASAS‐R	Sousa (Sousa et al., 2010), America	3, 15	Having power for self‐care, developing power for self‐care and lacking power for self‐care	Construct: three factors (61.7% explained variance) CFA with a good model fit (GFI, 0.942; CFI, 0.96; RMSEA, 0.05) Factor loading = 0.52–0.81	Internal consistency: Cronbach's *α* = 0.79–0.86 Inter‐item correlation:0.30–0.70 Item‐total correlation:0.30–0.71	Adult population

Abbreviations: ASAS‐R, Appraisal of Self‐Care Agency Scale‐Revised; BP, blood pressure; CFA, confirmatory factor analysis; CFI, Comparative Fit Index; DASH, Dietary Approaches to Stop Hypertension; GFI, Goodness of Fit Index; HPSMBRS, Hypertension Patients Self‐Management Behaviour Rating Scale; H‐SCALE, Hypertension Self‐Care Activity Level Effects; NFI, Normed Fit Index; RMSEA, root mean square error of approximation; SASE, Self‐Care Ability Scale for the Elderly.

Hence, this research aims to study the characteristics and application of these two hypertension self‐care instruments, test their reliability and validity and identify the best cut‐off points for SC‐HI and HBP SCP in a Chinese sample of older adults with hypertension, so as to give a reference for related future research in the Chinese context.

## 2 METHODS

### 2.1 Study design and participants

This is a cross‐sectional observational study. We enrolled participants at the Department of Cardiovascular Medicine of four tertiary referral centres from July–September 2019. The sample size was determined according to the maximum number of dimensions of the scales used. It was calculated by the formula in “Statistical Methods for Biomedical Research” edited by Jiqian Fang: sample size = [Max (number of dimensions) *(15–20)] *[1 + (15%–20%)] (Fang, 2007). The number of dimensions of the Exercise of Self‐Care Agency Scale (ESCA) in this study is the largest (4), so it was used as the benchmark for calculating the sample size (69–96 cases). Considering a 20% sample attrition rate, the final sample size of this study was 83–115 cases. Accordingly, the sample size should be enlarged as much as possible.

The inclusion criteria were patients who: (1) were aged 60 years or older (Liu et al., 2016), (2) had been diagnosed with hypertension by physicians, (3) were on antihypertensive medications, (4) were able to communicate without barriers and understand the questionnaires (Dickson et al., 2017; Han, Song, et al., 2014) and (5) could give written informed consent. Patients were excluded if they had acute or advanced diseases (such as cerebral infarction, acute myocardial infarction or advanced cancer), or if they had mental illness or any other severe disease. Using convenience sampling, 220 patients were recruited for this study. The patients who agreed to participate received a questionnaire covering sociodemographic and clinical information, the SC‐HI scale, the HBP SCP scale and the ESCA scale. According to patients' blood pressure value, as defined by the European Society of Hypertension (ESH), their hypertension was categorized into three stages: stage I hypertension (systolic BP [SBP] 140–159 mmHg and/or diastolic BP [DBP] 90–99 mmHg), stage II hypertension (SBP 160–179 mmHg and/or DBP 100–109 mmHg) and stage III hypertension (SBP≥180 mmHg and/or DBP≥110 mmHg) (Liu, 2011; Voulgaris et al., 2021). This study was approved by the Institutional Review Board of the hospital affiliated with Nantong University (Nantong University Ethical Review 2016‐K142).

### 2.2 Data collection

#### 2.2.1 Sociodemographic questionnaire

A self‐reported questionnaire that included demographic features (age, gender, education, former/present occupation, family income, marital status, socioeconomic status, history of drinking and smoking, housing conditions and primary caregivers) and clinical variables (height, weight, the highest and stable blood pressure level, duration of hypertension, and comorbidities and cardiovascular risk factors such as history of stroke, diabetes, history of myocardial infarction, dyslipidemia, etc.) was obtained by asking patients or viewing medical records.

#### 2.2.2 Questionnaires to measure Hypertension Self‐Care ability

##### Self‐Care of Hypertension Inventory

The SC‐HI, developed by Dickson et al. (2017), is one of the instruments used to measure the ability of self‐care in patients with hypertension. It is a self‐reporting scale consisting of 23 items divided across three subscales: self‐care maintenance, self‐care management and self‐care confidence. The items are related to behaviours used to maintain physical and emotional stability; the process of observing oneself for changes in signs and symptoms; responding to signs and symptoms; evaluating self‐care intervention effectiveness; confidence in the ability to perform a specific action; and the persistence in performing that action (Dickson et al., 2017). The SC‐HI has already been tested and used in Brazil and China (Silveira et al., 2018; Zhao et al., 2019). The internal consistency reliability coefficients of the self‐care maintenance and self‐care confidence scales were both 0.83. A multidimensional self‐care management factor included “consultative” and “autonomous” factors with a factor determinacy score of 0.75 (Dickson et al., 2017).

The SC‐HI comprises a Likert scale with items ranging from 1–4 (“never or rarely” to “always or daily”) in the self‐care maintenance domain, with two items ranging from 0–4 to capture a fully negative response: “I did not recognize it” to “very quickly” and “I did not try anything” to “very sure”; four items scored from 1–4 (“not quickly” to “very quickly”) in the self‐care management domain; and six items ranging from 1–4 (“not confident” to “extremely confident”) in the self‐care confidence domain. Each of the three scales is scored separately and standardized from 0–100, with higher scores indicating better self‐care. Additionally, self‐care is considered adequate if the separate score is 70 or greater (Silveira et al., 2018).

##### Hypertension Self‐Care Profile

The HBP SCP, developed by Han, covers three key domains of behaviour, motivation and self‐efficacy that can be used concurrently or independently. Han, Song, et al. (2014) used the HBP SCP‐Behaviour scale to create the motivation and self‐efficacy scales by changing the instructions from “How often do you do the following?” to “How important is it for you to do the following?” and “How confident are you that you could do the following?,” respectively. The HBP‐SCP scale consists of 60 items related to behaviour changes, medication adherence and lifestyle modifications. It has been tested and used in China and Singapore (Han, Song, et al., 2014; Ma et al., 2021; Seow et al., 2017; Sha et al., 2017). Item‐total correlations of the three subscales ranged from 0.20–0.74. The internal consistency reliability coefficients ranged from 0.83–0.93 (Han, Song, et al., 2014).

Each HBP SCP scale includes 20 items, rated using a 4‐point Likert scale ranging from 1 (*rarely/never*) to 4 (*always*), with higher scores indicating higher levels of HBP self‐care behaviour, motivation and self‐efficacy. The maximum score that could be obtained from this instrument is 240, which indicates the greatest degree of self‐care ability (Han, Song, et al., 2014; Ma et al., 2021; Sha et al., 2017).

##### Exercise of Self‐Care Agency Scale

The ESCA, developed by Kearney and Fleischer (Kearney & Fleischer, 1979), is a 43‐item instrument that measures an individual's self‐care ability. It has been cross‐culturally adapted into Chinese and validated in a sample of 284 women with chronic diseases (Guo et al., 2017; Wang & Laffrey, 2000). This is the most widely used measure in mainland China (Drevenhorn et al., 2015). The instrument consists of four dimensions: an active versus passive response to situations, motivation to care for oneself, knowledge base and self‐worth, which influences self‐esteem and supports health priorities (Drevenhorn et al., 2015). The content validity index was 1.0, internal consistency reliability coefficients ranged from 0.86–0.92, and the test–retest reliability coefficient was 0.91 after 1 week (Wang & Laffrey, 2000).

Each statement is rated on a 5‐point Likert scale, ranging from 0 (“*very uncharacteristic of me*”) to 4 (“*very characteristic of me*”). Eleven items (items 3, 6, 10, 16, 19, 22, 25, 28, 32, 34 and 39) are worded negatively with respect to exercise of self‐care, and a reverse scoring is used. The maximum score that can be obtained from the scale is 172, which indicates the greatest degree of self‐care ability. The higher the score, the higher the perceived self‐care ability (Istek & Karakurt, 2016). According to the scores of the total scale and subscales, the self‐care ability is divided into three levels: scores accounting for more than 66% of the total indicate a high level; scores accounting for 33%–66% of the total indicate a moderate level; and scores accounting for less than 33% of the total indicate a low level (Wang & Laffrey, 2000).

### 2.3 Data analysis

Data were analysed using the SPSS Version 20.0 and MedCalc 15.0. In the first instance, tests for normality were performed by implementing the Kolmogorov–Smirnov tests. A descriptive analysis was initially performed to investigate the participants' characteristics. Continuous data were presented as mean (standard deviation), categorical variables as frequencies and ranked data as frequencies.

The reliability of SC‐HI and HBP SCP was measured using Cronbach's *α* coefficients, the Guttman split‐half coefficients and item‐to‐total correlations in order to assess the internal consistency. A value of 0.7 was considered acceptable, while that above 0.8–0.9 was considered good to excellent internal consistency (Seow et al., 2017). A high Cronbach's *α* coefficient can reflect a high degree of homogeneity or internal consistency about the items (Tomstad et al., 2013).

Criterion validity is defined as the degree to which the scores of a test are an adequate reflection of the “gold standard” (Himuro et al., 2017). It was estimated using the Pearson's correlation coefficients between the total score of the SC‐HI and ESCA and their factors and between the total score of the HBP SCP and ESCA and their factors.

Exploratory factor analyses (EFA) were used to examine the construct validity of SC‐HI and HBP SCP. Factor analysis aimed to ascertain the number of latent variables that explain most of the variance in a given data set (Zhao et al., 2019).

Sensitivity, specificity, cut‐off points, positive predictive value (PPV) and negative predictive value (NPV) for the SC‐HI and HBP SCP were obtained through the Receiver Operating Characteristic (ROC) analysis (Martins et al., 2015; Tomstad et al., 2013), with a 95% confidence interval (95% CI) and a lower bound of >0.5 (Seule et al., 2016). The ESCA was regarded as the criterion. A total score of ≥66% on the ESCA shows a high degree of self‐care, and a total score of <66% suggests a middle‐low level of self‐care (Guo et al., 2017; Wang & Laffrey, 2000). The cut‐off point could be used to determine the utility of SC‐HI and HBP SCP scores for distinguishing non‐cases by checking the value of the Area under the curve (AUC) with the corresponding sensitivity and specificity. The Youden's index (J = sensitivity + specificity − 1), defined as the overall correct classification rate minus 1 at the cut‐off point (Yin & Tian, 2014), ranges from 0 to 1 (0 represents a complete overlap, 1 represents a complete separation) (Xu et al., 2014). We used the maximum of the Youden's index as the optimality criterion for cut‐off point selection in the ROC curve analysis, which could be calculated by identifying the point on the ROC curve nearest to the upper left‐hand corner (Bantis et al., 2014; Jorritsma et al., 2012; Nanishi et al., 2015). The AUC is a quantitative indicator of the information content of a test (Vivilaki et al., 2009), and it can give a reference of the measurement instrument's discrimination power, with values ranging from 0.5 (corresponding to a useless diagnostic test) to 1 (corresponding to a perfect diagnostic test) (Gengsheng & Hotilovac, 2008; Martins et al., 2015). Nanishi et al. (2015) proposed three categories: a test with an AUC greater than 0.90 indicates high accuracy, 0.7–0.9 indicates moderate accuracy, and 0.5–0.7 indicates low accuracy. All tests were two‐tailed, and a *p*‐value of <.05 was taken as statistically significant.

## 3 RESULTS

### 3.1 Sample characteristics

A total of 220 survey questionnaires were handed out and recovered on the spot, with a recovery rate of 100%. Among the included participants, 120 (54.55%) were male and 100 (45.45%) were female. Table 2 shows the sociodemographic and clinical characteristics of the participants.

**TABLE 2:** Sociodemographic and clinical characteristics of participants (*n* = 220)

Variables	*N* (%)
*Gender*	
Males	120 (54.55%)
Females	100 (45.45%)
Age, years, mean (range, SD)	73.74 (60–93 years, 8.97)
Duration of hypertension, mean (range, SD)	14.28 (1 month‐64 years, 11.57)
*Marital status*	
Single	1 (0.45%)
Married/stable union	168 (76.36%)
Separated/divorced	0 (0%)
Widower	51 (23.18%)
*Education level*	
Elementary or below	84 (38.18%)
Middle school	58 (26.36%)
High school	51 (23.18%)
College or above	27 (12.27%)
*Insurance*	
Yes	195 (88.64%)
No	25 (11.36%)
*Former/present occupation*	
Farmers	64(29.09%)
Workers	65 (29.55%)
Institution staff members	82 (37.27%)
Self‐employed households	9 (4.09%)
Others	0 (0%)
BMI, kg/m^2^; mean (range, SD)	24.20 (14.84–37.20, 3.82)
*Stage of blood pressure*	
Stage I hypertension	18 (8.18%)
Stage II hypertension	60 (27.27%)
Stage III hypertension	142 (64.55%)

Abbreviations: BMI, body mass index; SD, standard deviation.

The mean age and duration of hypertension were 73.74 (SD = 8.97) years (range, 60–93) and 14.28 (SD = 11.57) years (range, 1 month to 64 years), respectively. Eighteen respondents (8.18%) had stage I hypertension (SBP 140–159 mmHg and/or DBP 90–99 mmHg), 60 (27.27%) had stage II hypertension (SBP 160–179 mmHg and/or DBP 100–109 mmHg), and 142 (64.55%) had stage III hypertension (SBP ≥180 mmHg and/or DBP≥110 mmHg) (Voulgaris et al., 2021). Considering ESCA as a gold standard, 129 participants were defined as having a high level of self‐care, and 91 participants were defined as having a middle‐low level of self‐care.

### 3.2 Reliability analysis

The Cronbach's *α* coefficient of the SC‐HI was 0.858, the Guttman split‐half coefficient of the SC‐HI was 0.701, and the three dimensions of the SC‐HI yielded a Cronbach's *α* coefficient of 0.690, 0.703 and 0.891, respectively. The Cronbach's *α* coefficient of the HBP SCP was 0.953, the Guttman split‐half coefficient of the HBP SCP was 0.906, and the three dimensions of the HBP SCP yielded a Cronbach's *α* coefficient of 0.782, 0.932 and 0.917, respectively.

There are two sets of items (items 6 and 11; and items 1 and 18) that if deleted would improve the overall Cronbach's *α* for the SC‐HI (Table 3) and HBP SCP (Table 4), respectively. The item‐to‐total correlations of the SC‐HI ranged between *r* = .165 and *r* = .668, where the average correlation was *r* = .439 (Table 3), and the item‐to‐total correlations of the HBP SCP ranged between *r* = .061 and *r* = .749, where the average correlation was *r* = .523 (Table 4).

**TABLE 3:** Item‐to‐total score Pearson's rank correlation coefficients for SC‐HI (*n* = 220)

Items	*r*	Cronbach's *α* if item deleted
1. Check your blood pressure?	.446*	0.849
2. Eat lots of fruits and vegetables?	.165*	0.857
3. Do some physical activity?	.214*	0.857
4. Keep doctor or nurse appointments?	.408*	0.855
5. Eat a low‐salt diet?	.383*	0.85
6. Exercise for 30 min?	.328*	0.859
7. Take medicines as prescribed?	.212*	0.856
8. Ask for low‐salt items when eating out or visiting others?	.436*	0.854
9. Use a system to help you remember your medicines? For example, use a pill box or reminders	.313*	0.853
10. Eat a low fat diet?	.348*	0.852
11. Try to lose weight or control your body weight?	.302*	0.861
12. How quickly did you recognize that your blood pressure was up?	.510*	0.855
13. Reduce the salt in your diet	.645*	0.845
14. Reduce your stress level	.668*	0.846
15. Be careful to take your prescription medicines more regularly	.419*	0.855
16. Call your doctor/ nurse for guidance	.473*	0.853
17. How sure were you that the action helped or did not help?	.593*	0.851
18. Control your blood pressure?	.439*	0.853
19. Follow your treatment regimen?	.533*	0.848
20. Recognize changes in your health?	.606*	0.849
21. Evaluate changes in your blood pressure?	.663*	0.846
22. Take action that will control your blood pressure?	.508*	0.851
23. Evaluate how well an action works?	.476*	0.851
The average item‐to‐total score coefficients of SC‐HI	.439	

Abbreviation: SC‐HI, Self‐Care Hypertension Inventory.

* *p* < .01.

**TABLE 4:** Item‐to‐total score Pearson's rank correlation coefficients for HBP SCP (*n* = 220)

Items	*r*	Cronbach's *α* if item deleted
*How often do you do the following?*		
1. Take part in regular physical activity (e.g. 30 min of walking 4‐5times a week)?	.315*	0.954
2. Read nutrition facts label to check information on sodium content?	.457*	0.952
3. Replace traditional high‐salt foods (e.g. canned soups, Oodles of Noodles) with low‐salt products (e.g. homemade soups, fresh vegetables)?	.410*	0.953
4. Limit use of high‐salt condiments (e.g. ketchup)?	.426*	0.953
5. Eat less than one teaspoon of table salt per day (6 grams)?	.535*	0.952
6. Eat less foods that are high in saturated (e.g. red meat, butter) and trans fat (e.g. shortening, lard)?	.422*	0.952
7. Use broil, bake or steam instead of frying when cooking?	.325*	0.953
8. Read nutrition facts label to check information on saturated (e.g. butter, red meat) and trans fat (e.g. lard, shortening)?	.462*	0.952
9. Replace traditional high‐fat foods (e.g. deep fried chicken) with low‐fat products (e.g. baked chicken)?	.476*	0.952
10. Limit total calorie intake from fat (less than 65 grams) daily?	.503*	0.952
11. Eat five or more servings of fruits and vegetables daily?	.316*	0.953
12. Practice moderation in drinking alcohol daily (two glasses or less for men; one glass or less for women)?	.371*	0.953
13. Practice non‐smoking?	.338*	0.953
14. Check your blood pressure at home?	.454*	0.952
15. Forget to take your blood pressure medicine?	.208*	0.953
16. Forget to fill your prescriptions?	.236*	0.953
17. Keep your weight down?	.283*	0.953
18. Monitor situations that cause a high level of stress (e.g. arguments, death in the family) resulting in blood pressure elevation?	.061	0.954
19. Engage in activities that can lower stress (e.g. deep breathing, meditation)?	.403*	0.953
20. See a doctor regularly?	.391*	0.953
*How important is it to you to do the following?*		
21. Take part in regular physical activity (e.g. 30 min of walking 4–5 times per week)?	.470*	0.952
22. Eat less processed foods such as (e.g. canned or frozen foods, lunch meats)?	.592*	0.952
23. Read nutrition facts label to check information on sodium content?	.628*	0.952
24. Replace traditional high‐salt foods (e.g. canned soups, Oodles of Noodles) with low‐salt products (e.g. homemade soups, fresh vegetables)?	.682*	0.951
25. Limit use of high‐salt condiments (e.g. ketchup)?	.688*	0.951
26. Eat less than one teaspoon of table salt per day (6 grams)?	.706*	0.951
27. Eat less foods that are high in saturated (e.g. red meat, butter) and trans fat (e.g. lard, shortening)?	.676*	0.951
28. Use broil, bake or steam instead of frying when cooking?	.631*	0.952
29. Read food nutrition facts label to check information on saturated (e.g. butter, red meats) and trans fat (e.g. lard, shortening)?	.641*	0.952
30. Replace traditional high‐fat foods (e.g. deep fried chicken) with low‐fat foods (e.g. baked chicken)?	.749*	0.951
31. Limit total calorie intake from fat (less than 65 grams) daily?	.612*	0.952
32. Eat five or more servings of fruits and vegetables daily?	.613*	0.952
33. Practice moderation in drinking alcohol daily (two glasses or less for men; one glass or less for women)?	.585*	0.952
34. Practice non‐smoking?	.604*	0.952
35. Check your blood pressure at home?	.684*	0.951
36. Take your blood pressure medicine?	.487*	0.952
37. Get your prescriptions filled?	.429*	0.952
38. Keep your weight down?	.563*	0.952
39. Try to stay away from anything and anybody that causes stress?	.576*	0.952
40. See a doctor regularly?	.591*	0.952
*How confident are you that you could*		
41. Take part in regular physical activity (e.g. 30 min of walking 4–5 times per week)?	.436*	0.953
42. Eat less processed foods such as (e.g. canned or frozen foods, lunch meats)?	.645*	0.952
43. Read nutrition facts label to check information on sodium content?	.641*	0.952
44. Replace traditional high‐salt foods (e.g. canned soups, Oodles of Noodles) with low‐salt products (e.g. homemade soups, fresh vegetables)?	.697*	0.951
45. Limit use of high‐salt condiments (e.g. ketchup)?	.693*	0.951
46. Eat less than one teaspoon of table salt per day (6 grams)?	.713*	0.951
47. Eat less foods that are high in saturated (e.g. red meat, butter) and trans fat (e.g. lard, shortening)?	.614*	0.952
48. Use broil, bake or steam instead of frying when cooking?	.565*	0.952
49. Read nutrition facts label to check information on saturated (e.g. butter, red meats) and trans fat (e.g. lard, shortening)?	.605*	0.952
50. Replace traditional high‐fat foods (e.g. deep fried chicken) with low‐fat products (e.g. baked chicken)?	.699*	0.951
51. Limit total calorie intake from fat (less than 65 grams) daily?	.651*	0.952
52. Eat five or more servings of fruits and vegetables daily?	.546*	0.952
53. Practice moderation in drinking alcohol daily (two glasses or less for men; one glass or less for women)?	.390*	0.953
54. Practice non‐smoking?	.481*	0.952
55. Check your blood pressure at home?	.683*	0.951
56. Take your blood pressure medicine?	.462*	0.952
57. Get your prescriptions filled?	.496*	0.952
58. Keep your weight down?	.540*	0.952
59. Try to stay away from anything and anybody that causes any kind of stress?	.638*	0.952
60. See a doctor regularly?	.594*	0.952
The average item‐to‐total score coefficients of HBP SCP	.523	

Abbreviation: HBP SCP, Hypertension Self‐Care Profile.

* *p* < .01.

### 3.3 Validity analysis

Table 5 summarizes the Pearson's correlation coefficients between the SC‐HI total score, the HBP SCP total score and the ESCA total score, respectively. The results showed a strong correlation between the HBP SCP and ESCA, with a Pearson's correlation coefficient of 0.700 and a moderate correlation between the SC‐HI and ESCA, with a Pearson's correlation coefficient of 0.494.

**TABLE 5:** Criterion validity (measured using Pearson's correlation coefficient between SC‐HI, HBP SCP and ESCA, respectively (*n* = 220)

Scale correlation with ESCA	Pearson's correlation coefficients*
SC‐HI	0.494
HBP SCP	0.700

Abbreviations: ESCA, Exercise of Self‐Care Agency Scale; HBP SCP, Hypertension Self‐Care Profile; SC‐HI, Self‐Care Hypertension Inventory.

* All correlations were statistically significant (*p* < .001).

The results of the EFA of the SC‐HI and HBP SCP indicated that the Kaiser–Meyer–Olkin (KMO) was 0.782 and 0.888, and the Bartlett's Test of Sphericity was 1,419.226 and 9,866.815, respectively, with statistical significance (*p* < .01). Principal factor analyses showed the presence of 4, 1 and 1 latent factors with eigenvalues >1.00, explaining 63.619%, 42.642% and 65.136% of the total variance, in three dimensions of the SC‐HI, respectively. After principal factor analyses and Varimax orthogonal rotation, 7, 4 and 4 latent factors were extracted with eigenvalues >1.00, explaining 66.045%, 64.874% and 62.623% of the total variance, in three dimensions of the HBP SCP, respectively.

### 3.4 SC‐HI and HBP SCP ROC analysis

As a screening instrument, the overall accuracy of the SC‐HI can be described using the AUC. After analysing the data, it was found that the ROC AUC of the SC‐HI was 0.754 (95% CI: 68.9–81.9), higher than the line of no information (AUC = 0.5). After performing the calculation of sensitivity and specificity for the SC‐HI scale, the maximum of the Youden's index is 37.89, as can be seen in Table 6. The best cut‐off point was assessed to be 120.50 for the older patients with hypertension, with values above it indicating a higher level of self‐care ability. This was based on the values of sensitivity, specificity, PPV, NPV and the ROC curve. Furthermore, according to this cut‐off point, sensitivity and specificity values were 82.95% and 54.95%, respectively.

**TABLE 6:** Analysis of the Receiver Operating Characteristic (ROC) curves for the Self‐care of Hypertension Inventory (SC‐HI) And the Hypertension Self‐care Profile (HBP SCP) (*n* = 220)

Scales	Cut‐off point	Sensitivity (%)	Specificity (%)	The Youden's Index	PPV (%)	NPV (%)	AUC (95% CI)
SC‐HI	120.50	82.95	54.95	37.89	64.90	76.25	0.754 (68.9–81.9)
HBP SCP	169.50	79.07	75.82	54.89	79.07	75.82	0.838 (78.5–89.2)

Abbreviations: 95% CI, 95% confidence interval; AUC, Area under the curve; HBP SCP, Hypertension Self‐Care Profile; NPV, negative predictive value; PPV, positive predictive value; SC‐HI, Self‐Care Hypertension Inventory.

With regard to the HBP SCP scale, the AUC was 0.838, which was higher than 0.80. The result indicated that the HBP SCP gave significantly effective discrimination (95% CI: 78.5–89.2). The maximum of the Youden's index was 54.89, and the best cut‐off point was assessed to be 169.5, with values above it indicating a higher level of self‐care ability. This was based on the values of sensitivity, specificity, PPV, NPV and the ROC curve. At this cut‐off, sensitivity and specificity values were 79.07% and 75.82%, respectively. The ROC AUC for this cut‐off point is 0.838 (95% CI: 78.5–89.2), as can be seen in Table 6.

Furthermore, when comparing the ROC AUC of these two scales, the HBP SCP scale showed the larger AUC. The calculation indicated that the difference between these two scales (SD = 0.030) was statistically significant. The data used for the prediction of a cut‐off value are presented in Table 6.

## 4 DISCUSSION

This study was the first to compare two instruments for hypertension self‐care measurements among older adults in China. The overall objective was to identify suitable instruments for use in nursing research and practice. The specific aim was to test and compare the reliability and validity of the Chinese versions of two hypertension self‐care instruments, the SC‐HI and HBP SCP, and identify the best cut‐off points for these two instruments among older adults.

In this study, the participants in the sample were from several hospitals, with demographic aspects such as age, gender, occupation and education level widely distributed. Thus, the sample had good representation, indicating a heterogeneous sample. Using the ESCA as an assessment tool among the participants recruited in this study, 129 (58.6%) and 91 (41.4%) were defined as having a high and middle‐low level of self‐care, respectively. Consistent with Tan et al.'s (2021) research, patients with high self‐efficacy were more likely to adhere to self‐care in their management of hypertension. Participants with stage III hypertension and a long duration of hypertension had better self‐care ability than other participants. A possible explanation is that participants with stage III hypertension would have several complications that affect their quality of life, necessitating strict regulation of their diet, activities and emotions to control diseases. In accordance with Wee et al.'s (2021) research, one of the common factors influencing higher scores was the level of education. The longer the duration of hypertension, the more health education the participant received. Hence, those who have had hypertension for a long time will acquire more health‐related knowledge. Future interventions to improve self‐care among patients with hypertension may need to be tailored to their behaviour, motivation and self‐efficacy levels (Wee et al., 2021).

Based on the results presented above, we found that the SC‐HI and HBP SCP scales presented adequate power in measuring self‐care ability when compared with the ESCA scale in the sample of older adults with hypertension. About internal consistency, the results showed that the Chinese versions of both the SC‐HI and HBP SCP could be considered reliable instruments. In accordance with previous reports, Cronbach's *α* coefficients of these two scales were both within the recommended standard (>0.70) and reflected sufficient homogeneity in the Chinese versions (Chen et al., 2014; Dickson et al., 2017; Han, Song, et al., 2014; Koh et al., 2016; Ma et al., 2021; Seow et al., 2017; Sha et al., 2017). Except for two sets of items (items 6 and 11; and items 1 and 18) of the SC‐HI and HBP SCP, respectively, the item‐specific analysis showed that item‐to‐total correlations were in agreement with the recommended standard (Wang & Laffrey, 2000). This may be related to patients' differing concepts about each dimension. These analyses showed that the SC‐HI and HBP SCP are both credible scales with a high level of consistency, and that the HBP SCP is slightly stronger than the SC‐HI in the present study.

This study gave evidence of the criterion validity of the Chinese versions of the SC‐HI and HBP SCP. The results indicated that the Pearson's correlation coefficient between the SC‐HI and ESCA was weaker compared with the Pearson's correlation coefficient between the HBP SCP and ESCA. A possible explanation was that the SC‐HI scale included the evaluation of self‐care intervention effectiveness, but the other two scales (HBP SCP and ESCA) did not.

Construct validity tested by EFA revealed the presence of four factors: exercise (items 3, 5, and 6), alimentary control (items 2 and 10), medication adherence (items 7 and 9) and symptom control (items 1, 4, 8, and 11) in the self‐care maintenance scale and a single factor in the other two subscales. These findings suggest that the self‐care maintenance scale is a multidimensional construct and that the other two subscales are unidimensional instruments. The findings are consistent with Dickson et al. (2017) and Silveira et al. (2018). The results related to the principal factor analyses of the HBP SCP confirmed the presence of seven factors including alimentary control (items 3, 4, 5 and 6), knowledge and behaviour (items 2, 8 and 19), medication adherence (items 15 and 16), emotion management (items 7, 18 and 20), alcohol and tobacco control (items 12 and 13), low‐fat diet (items 9, 10 and 11) and symptom control (items 1, 14 and 17) in the behaviour scale; four factors including alimentary control (items 4, 5, 6, 7 and 12), symptom control (items 3, 8, 9, 10, 11, 15, 18, 19 and 20), alcohol and tobacco control and medication adherence (items 13, 14, 16 and 17) and exercise (items 1 and 2) in the motivation scale; and four factors including alimentary control (items 2, 4, 5, 6, 7, 8, 10, 11 and 12), symptom control (items 1, 15, 16, 17 and 18), knowledge and emotion management (items 3, 9, 19 and 20) and alcohol and tobacco control (items 13 and 14) in the self‐efficacy scale. The EFA results of the HBP SCP were slightly different from Mas research. This may be because the surveyed population was different (Ma et al., 2021). The target participants in Ma et al.'s research were adults with hypertension. However, this study was conducted in the older hypertensive population.

The results of the ROC curves showed that the AUC of the HBP SCP was larger than that of the SC‐HI in this study. The reasons for this result could be twofold: first, the items of the HBP SCP may be easier to understand for older patients with hypertension than the SC‐HI. Second, the SC‐HI management scale is only completed by patients who report that their blood pressure has been high in the previous interval. Therefore, it may have led to different results. Accordingly, the investigation results of the HBP SCP were better than the results of SC‐HI.

This study is the first to explore the cut‐off points of the SC‐HI and HBP SCP. The cut‐off point was determined using an interpretation of the best values for sensitivity, specificity, PPVs and NPVs. The results of ROC analysis indicated that the sensitivity, specificity, Youden's index and AUC are all acceptable. In this sense, according to the ROC AUC, this study contributed by noting that, when taking the effectiveness of scales into consideration, both the SC‐HI scale and the HBP SCP scale presented themselves as good instruments for measuring the ability of self‐care in older patients with hypertension.

It is integral that addressing gaps in self‐care measurement requires that instruments reflect current clinical guidelines and advances in the theory upon which they have been developed (Dickson, 2021). About the characteristics of these two scales, the SC‐HI was conceptualized by the middle range theory of self‐care in chronic illness. It covers a range, such as the decision‐making process, self‐care monitoring and the evaluation of self‐care intervention effectiveness, emphasizing the importance of observing oneself for changes in signs and symptoms (Dickson, 2021; Dickson et al., 2017). The HBP SCP was developed based on Orem's self‐care theory and explored behaviour, motivation and self‐efficacy (Han, Lee, et al., 2014). The items in the HBP SCP scale and the SC‐HI scale all focus on the assessment of personal self‐care skills. Nevertheless, different characteristics, advantages and disadvantages can be found between these two scales. To a certain extent, both are complementary, because all their subscales can be used separately or combined. The SC‐HI could evaluate the effectiveness of the self‐care intervention. When necessary, it can effectively compensate for the deficiency of the HBP SCP in the clinical selection or future research. However, to ensure the accuracy of the survey, it is best not to use these two scales in combination all the time because of the large number of items. Nevertheless, researchers can choose the optimal scale to use according to the characteristics of participants and situations.

## 5 LIMITATIONS AND FUTURE RESEARCH

Since the SC‐HI management scale was only completed by patients who reported having high blood pressure in the previous interval, different findings may have been obtained, which can be a potential limitation. In follow‐up studies, more participants should be recruited to avoid such influences on the study results.

## 6 CONCLUSION

The SC‐HI and HBP SCP are both credible instruments for the self‐care evaluation of older adults with hypertension in China. In conclusion, this study can not only give impetus for the development of hypertension self‐care assessment tools but also give assistance in selecting the appropriate scale for future studies. However, further studies should be conducted to test the reliability, validity, sensitivity and specificity in other hypertensive groups with larger sample sizes in China.

## 7 RELEVANCE TO CLINICAL PRACTICE

Both the SC‐HI and the HBP SCP are valid instruments to measure the self‐care ability of older adults with hypertension in China and presumably in other elderly populations too. Furthermore, they may give an impetus for the development of other hypertension self‐care assessment tools. In addition, future researchers have a basis for selecting appropriate scales for them to begin with.

## AUTHOR CONTRIBUTIONS

All listed authors meet the authorship criteria, and they are in agreement with the content of the manuscript. Qiao Zhao analysed the data and drafted the entire manuscript. Yujie Guo designed the study. Lipei Gu and Lei Yang collected the data. There is no financial conflict in the information contained in this manuscript.

## FUNDING INFORMATION

Natural Science Foundation of Jiangsu Province (BK 20191447).

## CONFLICT OF INTEREST

The authors have no conflicts of interest to disclose.

## Data Availability

The data that support the findings of this study are available from the corresponding author upon reasonable request.
